# Fractional-order modeling and optimal control of lassa fever with non-pharmaceutical interventions

**DOI:** 10.1016/j.mex.2026.103932

**Published:** 2026-04-30

**Authors:** Akeem Olarewaju Yunus, Oludolapo Akanni Olanrewaju

**Affiliations:** Institute of Systems Science (ISS), Durban University of Technology, Durban. South Africa

**Keywords:** Atangana–baleanu–caputo derivative, Epidemic control, Non-pharmaceutical interventions, Lassa fever, Memory-dependent dynamics, Resource optimization

## Abstract

This paper presents a fractional-order model of Lassa fever transmission by applying the Atangana Baleanu Caputo (ABC) derivative which include the memory and hereditary effects in the disease dynamics. Human and rodent populations in the model, with non-pharmaceutical interventions, such as public enlightenment and quarantine as time-dependent control variables. Qualitative properties positivity, invariant region, equilibrium states, and the basic reproduction number R_0_ are strictly examined. The disease-free and endemic equilibria are determined as stable with the help of the Lyapunov methods. A control framework is developed to optimize the costs of intervention and the infection burden and the conditions are obtained through the Maximum Principle of Pontryagin. Numerical simulations, performed through the Laplace Adomian Decomposition Method, illustrate the influence of memory effects in disease persistence and that combined control measures are effective in reducing the level of infection, length of an outbreak and the cost of the same. Sensitivity analysis determines transmission and intervention parameters to be important contributors of R_0_. The findings emphasize the significance of awareness and the need to continue with quarantine at the early stage, and indicate the usefulness of the fractional-order modeling as a powerful tool to in the effective and resource-efficient epidemic control strategies.

## Graphical abstract

**Figure:**
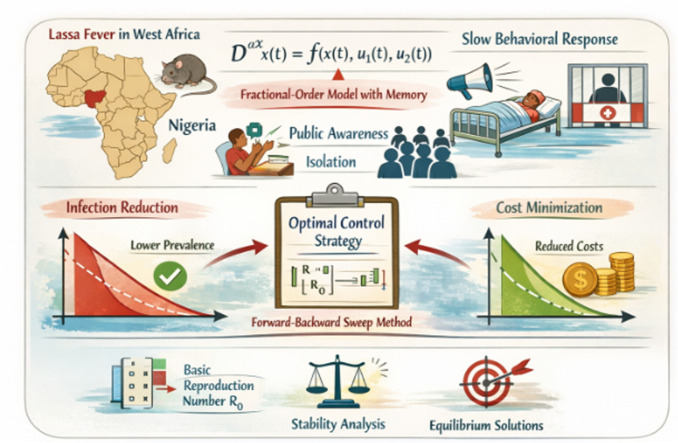

## Introduction

Infectious disease outbreaks present not only public health challenges but also complex control and optimization problems, particularly in resource-limited settings where intervention capacity, timing, and compliance critically influence outcomes [[1], [2], [3], [4], [5]]. West Africa's continent-wide Lassa fever is a zoonotic transmissible hemorrhagic disease that still causes significant morbidity .mortality, and economic burden, with Nigeria bearing the highest disease incidence and recurrent seasonal outbreaks [[6], [7], [8], [9], [10]]. Despite sustained public health efforts, effective containment remains challenging due to delayed behavioral responses, imperfect intervention enforcement, and persistent animal reservoirs.

From a systems and control perspective, Memory-dependent phenomena, such as delayed public knowledge acquisition, intrinsically affect the dynamics of Lassa fever propagation, gradual compliance with preventive measures, and lagged quarantine responses [11,[15], [16], [17]]. Classical integer-order epidemic models assume instantaneous system responses and thus fail to adequately represent these nonlocal temporal effects. As a result, such models may underestimate the long-term impact of interventions and provide limited guidance for optimal policy design [18,24].

Fractional-order dynamical systems have become an effective modeling paradigm to describe the memory and hereditary effects of complex systems. Fractional derivatives are used to model past conditions of a system in present dynamics in epidemiological modeling to provide a more lifelike view of disease transmission and intervention impacts [13,14,19,[22], [26], [27], [28], [29], [30]]. Specifically, the Atangana Baleanu Caputo (ABC) fractional derivative which possesses a non-singular Mittag Leffler kernel has been found to be more numerically stable and physically interpretable than the classical Caputo and Caputo Fabrizio derivatives [12,[32], [33], [34]]. It is this combination of properties that make the ABC operator particularly appropriate to controlled epidemiological systems, in which past intervention activities still affect the current disease dynamics.

A number of mathematical models have been created to examine the dynamics of Lassa fever transmission, with environmental transmission, rodent reservoirs, treatment strategies, and control measures [7,[18], [19], [20], [21], [22], [23], [24], [25]]. While these studies have significantly advanced understanding of disease spread, most treat non-pharmaceutical interventions—such as quarantine enforcement and public awareness campaigns—as fixed parameters. This modeling choice limits their applicability for policy formulation, as real-world interventions are inherently time-dependent decisions constrained by resources and costs [11,25,31].To model metabolic memory effects, [[35], [36]] constructs a Caputo Fabrizio fractal-fractional glucose insulin model with constant and variable orders, and employs a stable Newton interpolation scheme to accurately simulate it. It recreates realistic glucose insulin oscillations, demonstrates feedback stabilization of chaotic behaviour, and can be used to support adaptive personalized diabetes control via clinically calibrated simulations. A fractal-fractional glucose-insulin model introduced in [37] that incorporates the dynamics and kinetics of the beta-cell, as well as the therapy effect, is simulated with the Adomian decomposition method to give stable, convergent, and accurate results of the dynamics of the diabetes system [38] Constructs an Atangana Baleanu fractal-fractional glucose insulin model that is proved to be stable with linear control, which enhances the precision of simulation and stabilization of the dynamics of diabetes [39] Builds an ABC fractal-fractional pneumonia model that has memory effects, demonstrating stability and enhanced simulation accuracy in disease prediction and control planning [40] Develops improved Newton polynomial-based numerical schemes for fractal–fractional Burke–Shaw systems, proving existence and uniqueness, computing Lyapunov exponents, and demonstrating accurate, efficient simulations consistent with analytical results.In control theory, intervention measures are naturally formulated as control variables acting on system dynamics. The epidemiological consequences and costs of implementation can be balanced by modeling the problem of quarantine and public enlightenment as a controlled, time-dependent response, making it possible to determine the optimal control problem. This approach is directly correlated with the scope of Results in Control and Optimization that focuses on decision-oriented modelling, system optimization, and control methods saving resources.

Based on these reasons, this paper will come up with a fractional-order controlled epidemic model of the Lassa fever based on the Atangana Baleanu Caputo derivative. Enlightenment and quarantine policies among the people are explicitly modeled as admissible control functions that can act on the dynamics of transmission and progression. Fractional optimal control problem is developed to minimize the burden of infection taking into consideration the cost of intervention. Such analytical properties of the controlled system as positivity, invariant regions, equilibrium stability, and the basic reproduction number are rigorously studied in the presence of memory effects. One of the work's main accomplishments is the development of a controlled dynamical system model for fractional-order Lassa fever that takes into account memory effects. The study also intends to establish a paradigm for fractional optimum control in non-pharmaceutical therapies by exploring reproduction dynamics and equilibrium stability in the presence of fractional memory. Numerical simulations are used to objectively assess the most effective intervention strategies and policy outcomes. The research paradigm suggested offers a mathematically sound and decision-making method of distributing scarce public health resources in the areas where the Lassa virus is common. Other infectious diseases having intervention-based dynamics and delayed behavioral response can also be studied using the modelling technique. This study contributes by developing a memory-dependent fractional-order Lassa fever model incorporating optimal control strategies. It delivers rigorous analytical outputs, such as stability and reproduction dynamics and provides quantitative information of effective intervention policies by the use of numerical simulation and sensitivity analysis.

## Preliminary

A basic method for fractional operators of the Atangana-Baleanu Caputo (ABC) derivative is presented in this section.

Definition 2.1 [28] ABC with $\eta \in \;\left[0,1\right],\;\;\forall p\left(t\right)\in P^{\prime }\left[0.T\right]$ ${}^{ABC}D_{t}^{\eta }p\left(t\right)=\frac{ABC}{\left(1-\eta \right)}\int_{0}^{t}E_{\eta }\left[\frac{-\eta }{1-\eta }{\left(t-w\right)}^{\eta }\right]\frac{d}{dw}p\left(w\right)dw$ Where $ABC\left(\eta \right)=\frac{\eta }{2-\eta }$ ABC(0)=ABC(1)=1

$E_{\eta }\left(x\right)=\sum_{h}^{\infty }\frac{x^{h}}{\Gamma (\eta h+1)}.$ (Mittag-Leffler function)

Definition 2.2 [28] Let 0≤η≤1and $p\left(t\right)\in L^{I}\left[0,T\right],$ in ABC as

$${}^{ABC}D_{t}^{t}p\left(t\right)=\frac{1-\eta }{ABC(\eta )}p\left(t\right)+\frac{\eta }{ABC(\eta )\Gamma (\eta )}\int_{0}^{t}p(w){\left(t-w\right)}^{t-1}dw.$$

Lemma 2.3

$$\left\{ \begin{array}{c} {}^{ABC}D_{0}^{t}p\left(t\right)=X\left(t\right),\\ X\left(0\right)=X_{0} \end{array}\right.$$

We get its solution as.

$$p\left(t\right)=p\left(0\right)+\frac{1-\eta }{ABC(\eta )}p\left(t\right)+\frac{\eta }{ABC(\eta )\Gamma (\eta )}\int_{0}^{t}p(w){\left(t-w\right)}^{t-1}dw.$$

Let ${}_{t}^{ABC}D^{\eta }p_{i}\left(t\right)=Z_{i}\left(\Omega_{1},\Omega_{2},\Omega_{3},\cdots \Omega_{i}\right)+Y_{i}\left(\Omega_{1},\Omega_{2},\Omega_{3},\cdots \Omega_{i}\right).$be a function

Implies$D_{i}^{\vartheta }\left(0\right)=\varsigma_{k}^{i}$, ∀i=1,2,3...m, $\;n_{i-1}\leq \vartheta \leq n_{i}$.

$${}_{t}^{ABC}D^{\eta }p_{i}\left(t\right)=Z_{i}\left(\Omega_{1},\Omega_{2},\Omega_{3},\cdots \Omega_{i}\right)+Y_{i}\left(\Omega_{1},\Omega_{2},\Omega_{3},\cdots \Omega_{i}\right).$$

$$Z_{i}\left(\Omega_{1},...\Omega_{i}\right)=\sum_{i=0}^{\infty }F_{ij}\left(t\right),\;i=1,2,...m$$

$$L\left[\sum_{j=0}^{\infty }\Omega_{ij}\left(t\right)\right]\;=\frac{\Omega_{i}^{k_{i}}\left(0\right)}{\varepsilon }+\frac{\varphi +\eta \left(1-x\right)}{\varepsilon }\left(L\;\;\left[L_{i}\left(\sum_{j=0}^{\infty }\Omega_{1j}\left(t\right),...,\sum_{j=0}^{\infty }\Omega_{mj}\left(t\right)\right)\right]+L\left[\sum_{j=0}^{\infty }M_{ij}\left(t\right)\right]\right).$$

$$\Omega_{i(j+1)}\left(t\right)=m_{k}^{i}+L^{-1}\left(\frac{\varphi +\eta \left(1-\varphi \right)}{\varepsilon }L\left[\;\;L_{i}\left(\sum_{j=0}^{\infty }p_{1j}\left(t\right),...,\sum_{j=0}^{\infty }p_{mj}\left(t\right)\right)\right]+L\left[\sum_{j=0}^{\infty }C_{ij}\left(t\right)\right]\right).$$

## Methods

### Formulation of the Lassa fever model

An extended SEIR-type framework is proposed to model Lassa fever transmission between human and rodent populations, where humans are grouped into susceptible, exposed, infectious, quarantined, and recovered classes, and rodents into susceptible and infectious compartments, building on earlier studies [7]. Public enlightenment and quarantine are introduced as explicit intervention processes that attenuate transmission and isolate infectious individuals, respectively, and are assumed to operate with memory-dependent characteristics.ABC fractional sense ensuring non-negativity of all state variables under biologically admissible initial conditions.

(1) $$\begin{array}{c} {S^{\prime }}_{H}\left(t\right)=\Lambda_{H}-S_{H}\left(1-\in \right)\left(\gamma I_{H}+aI_{R}\right)-\mu_{H}S_{H}+\tau R_{H},\\ {E^{\prime }}_{H}\left(t\right)=S_{H}\left(1-\in \right)\left(\gamma I_{H}+aI_{R}\right)-\left(\varphi +\mu_{H}\right)E_{H},\\ {I^{\prime }}_{H}\left(t\right)=\varphi \;{E^{\prime }}_{H}\left(t\right)-\left(\delta +\theta +\mu_{H}\right)I_{H},\\ {Q^{\prime }}_{H}\left(t\right)=\theta I_{H}-\left(\omega +\mu_{Q}\right)Q_{H},\\ {R^{\prime }}_{H}\left(t\right)=\delta I_{H}-\omega Q_{H}-\left(\tau +\mu_{H}\right)R_{H}\\ {S^{\prime }}_{R}\left(t\right)=\Lambda_{R}-\rho S_{R}I_{R}-\mu_{R}S_{R},\\ {I^{\prime }}_{R}\left(t\right)=\rho S_{R}I_{R}-\mu_{R}I_{R}. \end{array}$$

The Lassa Fever Model's Assumptions It is assumed that all parameters and state variables are positive. Everybody is susceptible to Lassa fever. An equal number of susceptible and infected people are thought to exist. Some people who have recovered might rejoin the susceptible group. Regardless of age or health, everyone is equally susceptible to Lassa fever.

(2) $$\begin{array}{c} {}^{ABC}D{}_{t}^{\eta }S_{H}\left(t\right)=\Lambda_{H}-S_{H}\left(1-\in \right)\left(\gamma I_{H}+aI_{R}\right)-\mu_{H}S_{H}+\tau R_{H},\\ {}^{ABC}D{}_{t}^{\eta }E_{H}\left(t\right)=S_{H}\left(1-\in \right)\left(\gamma I_{H}+aI_{R}\right)-\left(\varphi +\mu_{H}\right)E_{H},\\ {}^{ABC}D{}_{t}^{\eta }I_{H}\left(t\right)=\varphi {E^{\prime }}_{H}\left(t\right)-\left(\delta +\theta +\mu_{H}\right)I_{H},\\ {}^{ABC}D{}_{t}^{\eta }Q_{H}\left(t\right)=\theta I_{H}-\left(\omega +\mu_{Q}\right)Q_{H},\\ {}^{ABC}D{}_{t}^{\eta }R_{H}\left(t\right)=\delta I_{H}-\omega Q_{H}-\left(\tau +\mu_{H}\right)R_{H,}\\ {}^{ABC}D{}_{t}^{\eta }S_{R}\left(t\right)=\Lambda_{R}-\rho S_{R}I_{R}-\mu_{R}S_{R},\\ {}^{ABC}D{}_{t}^{\eta }I_{R}\left(t\right)=\rho S_{R}I_{R}-\mu_{R}I_{R}. \end{array}$$

With initial condition.

$${S_{H}}_{0}=J_{1},{E_{H}}_{0}=J_{2},{I_{H}}_{0}=J_{3},{Q_{H}}_{0}=J_{4},R_{H0}=J_{5}{S_{R}}_{0}=J_{6},{I_{R}}_{0}=J_{7}.$$

Where ${}^{ABC}D^{\eta }0\leq \eta \leq 1$ ABC derivative with η order of derivative

In model (2), the initial conditions are no reliant and meet a specific relation and more so, the initial conditions play a critical role in the description of the behavior of the system. Fractional order model solutions that are semi-analytical are investigated using an Adomian Decomposition Method (ADM) of the Laplace transformation. The current approach presents the dynamic of the system in a detailed analysis and description of the parameters in Table 1 below. To verify the accuracy of the results we have random numbers on initial conditions and parameters where the numerical solutions are sound and strong.

**Table 1:** Estimated parameter values for lassa fever model validation.

Parameter	Description	Estimated Value with unit	Source / Assumption
γ	Contact rate of human	0.002 per contact	Estimated from Nigeria outbreak data (NCDC, 2025)
$\mu_{H}$	Natural mortality rate	0.000045 day⁻¹	Approx. life expectancy in Nigeria (2024)
δ	Disease-induced mortality rate	0.015 day⁻¹	NCDC outbreak fatality reports
θ	Rate of progression from exposed to infectious	1/7 day⁻¹	Average incubation period of 7 days (WHO, 2025)
Λ	Recruitment rate	0.00012 day⁻¹	Derived from Nigeria’s crude birth rate
ε	Effectiveness of awareness/behavioral interventions	0.3 day⁻¹	Logical assumption based on reported intervention impacts
∈	Level of public enlightenment	0.2 day⁻¹	Assume
θ	Quarantine rate of infectious individuals	0.5 day⁻¹	Assume
ω	Recovery rate in quarantine	0.5 day⁻¹	Assume
$\mu_{Q}$	Natural death rate in quarantine	0.000045 day⁻¹	Approx. life expectancy in Nigeria (2024)
$\Lambda_{H}$	Humans are of recruitment	20,000 persons/day	[8]
$\Lambda_{R}$	Recruitment rate for rodent	200 day⁻¹	Assume
a	Contact rate of rodent.	0.4 day⁻¹	Assume
φ	Rate of Progression to the infectious class.	0.001 day⁻¹	Assume
ρ	Rate of immunity lost.	0.1 day⁻¹	Assume
τ	waning immunity rate	0.2 day⁻¹	Assume

### Model analysis

#### Positivity of solutions

To make sure the model makes sense from an epidemiological point of view, we check that every state variable stay positive for allt>0, given that the starting conditions are also positive. Think about the fractional Caputo– in the form of the Atangana–Baleanu operator: ${}^{ABC}D_{t}^{\eta }ℵ\left(t\right)=p\left(ℵ\left(t\right)\right)\geq 0$Since each equation in the model contains non-negative inflows and outflows, and the Caputo– Atangana–Baleanu operator preserves positivity the solutions $S_{H}\left(t\right),E_{H}\left(t\right),\;I_{H}\left(t\right),\;Q_{H}\left(t\right),R_{H}\left(t\right),\;S_{R}\left(t\right),I_{R}\left(t\right)$≥0 for all t>0.

#### Invariant region

Let $S_{H}\left(t\right)+E_{H}\left(t\right)+I_{H}\left(t\right)+Q_{H}\left(t\right)+R_{H}\left(t\right)+S_{R}\left(t\right)+I_{R}\left(t\right)$. Summing the model equations yields:

(3) $${}^{ABC}D_{t}^{\alpha }N\left(t\right)=\Lambda_{H}+\Lambda_{R}-\left(\mu_{H}+\mu_{R}\right)N\left(t\right).$$

Solving this linear fractional differential equation gives:

$N\left(t\right)\to \frac{\Lambda_{H}+\Lambda_{R}}{\left(\mu_{H}+\mu_{R}\right)}$ Thus, the region

(4) $$ℵ=\left\{\left(S_{H},E_{H},\;I_{H},\;Q_{H},R_{H},\;S_{R},I_{R}\right)\in R^{+7}|N\left(t\right)\to \frac{\Lambda_{H}+\Lambda_{R}}{\left(\mu_{H}+\mu_{R}\right)}\right\}.$$

remains positive and has a limit. So, the model is well-posed and possible in biology.

#### Disease-free equilibrium (DFE)

In this equilibrium state of the model, disease is considered to be absent thus$E_{H}=I_{H}=Q_{H}=I_{R}=0$. Solving the model at this state, we obtain:

(5) $$\begin{array}{c} S_{H}^{*}=\frac{\Lambda_{H}}{\mu_{H}},\;\;S_{R}^{*}=\frac{\Lambda_{R}}{\mu_{R}},\\ \varepsilon_{0}=\left(S_{H}^{*},\;\;\;E_{H}^{*},\;\;I_{H}^{*},\;\;Q_{H}^{*},\;R_{H}^{*},\;\;S_{R}^{*},\;\;\;I_{R}^{*}\right).=\left(\frac{\Lambda_{H}}{\mu_{H}},\;\;\;0,\;\;0,\;\;0,\;0,\;\;\frac{\Lambda_{R}}{\mu_{R}},\;\;\;0\right). \end{array}$$

#### **Basic reproduction number**$R_{0}$

The fundamental reproduction number using the next-generation matrix method is: Select the vector of the infected state.

$x={\left[E_{H},I_{H},Q_{H},I_{R}\right]}^{T}$. Split the dynamics as

New infections F (linearized at $E_{0}$):

(6) $$F=\left(\begin{array}{cccc} 0 & \left(1-\varepsilon \right)a\frac{\Lambda_{H}}{\mu_{H}} & \left(1-\varepsilon \right)\gamma \frac{\Lambda_{H}}{\mu_{H}} & 0\\ 0 & 0 & 0 & 0\\ 0 & 0 & 0 & 0\\ 0 & 0 & 0 & \rho \frac{\Lambda_{R}}{\mu_{R}} \end{array}\right)$$

Transitions V (outflow − inflow not due to new infection), using the usual progression/removal rates (exposed to infectious, infectious to recovery and quarantine, quarantined to recovery):

(7) $$V=\left(\begin{array}{cccc} \varphi +\mu_{H} & 0 & 0 & 0\\ -\varphi & \delta +\theta +\mu_{H} & 0 & 0\\ 0 & -\theta & \omega +\mu_{Q} & 0\\ 0 & 0 & 0 & \mu_{R} \end{array}\right).$$

Spectral radius of $FV^{-1}$ because the matrix is block upper‑triangular, $R_{0}$decomposes into:

(8) $$R=\left(1+\varepsilon \right)\frac{\Lambda_{H}}{\mu_{H}}\frac{\varphi }{\left(\varphi +\mu_{H}\right)\left(\delta +\theta +\mu_{H}\right)}\frac{\rho \Lambda_{R}}{\mu_{R}}.$$

(If quarantine does not return to infectiousness, ω does not enter $R_{0}$ directly; it improves outcomes by shortening infectious time via θ moving people out of $I_{H}$. If your specific model allows other couplings, adjust the corresponding entries of F and V.) Interpretation: the epidemic can invade the human population if $R_{H}>1$, or sustain in the rodent population if $R_{R}>1$. Cross–species infection (rodent human) cannot by itself create a spectral radius exceeding the larger of the two diagonal reproduction numbers because of the triangular structure — coexistence dynamics depend on which diagonal block exceeds 1.

#### Local stability of disease free equilibrium

${}^{ABC}D_{t}^{\eta }x=Jx$, the equilibrium is locally asymptotically stable iff every eigenvalue $\lambda_{i}\left(J\right)$ satisfies

(9) $$\left|\arg(\lambda_{i}\left(J\right))\right|>\eta \pi /2.$$

For epidemiological systems written via the next‑generation approach, a sufficient and standard condition is:

(10) $$R_{0}<1\;\;\;\;\Rightarrow \varepsilon_{0}\;\;\;\text{is}\text{locally}\text{asymptotically}\text{stable}\text{for}\;\eta \;\;\;\in \left(0,1\right]$$

If any transmission terms displayed equations differ additional shedding to the reservoir or feedback from $Q_{H}$), then corresponding closed‑form $R_{0}$.

#### Endemic equilibrium

The proposed endemic equilibrium is a stable point where the disease stays in the population and becomes a normal part of life. The endemic balance $\varepsilon_{*}=\left({S_{H}}_{*},{E_{H}}_{*},{I_{H}}_{*},{Q_{H}}_{*},{R_{H}}_{*},{S_{R}}_{*},{I_{R}}_{*}\right)$ occurs when all derivatives set to Zero and solving the algebraic system.

Rodent subsystem.

(11) $$\begin{array}{c} \Lambda_{R}-\rho S_{R}^{*}I_{R}^{*}-\mu_{R}S_{R}^{*}=0,\\ \;\;\;=I_{R}^{*}\left(\rho S_{R}^{*}-\mu_{R}\right)=0. \end{array}$$

Since endemicity requires $I_{R}^{*}>0$ then $S_{R}^{*}=\frac{\mu_{R}}{\rho }$ .

Substituting $S_{R}^{*}$ into the Eq. (11) gives

$$\;\;I_{R}^{*}=\frac{\Lambda_{R}}{\mu_{R}}-\frac{\mu_{R}}{\rho }.$$

Thus endemic rodent infection exists whenever $R_{R}=\frac{\rho \Lambda_{R}}{{\mu^{2}}_{R}}>1$

For human subsystem.

(12) $$\begin{array}{c} \Lambda_{H}-\lambda^{*}S_{H}^{*}-\mu_{H}S_{H}^{*}+\tau R_{H}^{*}=0,\\ \lambda^{*}S_{H}^{*}-\left(\varphi +\mu_{H}\right)E_{H}^{*}=0,\\ \varphi E_{H}^{*}\left(t\right)-\left(\delta +\theta +\mu_{H}\right)I_{H}^{*}=0,\\ \theta \;I_{H}^{*}-\left(\omega +\mu_{Q}\right)Q_{H}^{*}=0,\\ \delta \;I_{H}^{*}-\omega Q_{H}^{*}-\left(\tau +\mu_{H}\right)R_{H}^{*}=0. \end{array}$$

where the endemic force of infection is $\lambda^{*}=\left(1-\in \right)\left(\gamma I_{H}^{*}+aI_{H}^{*}\right)$

Now expressing Compartments in terms of $I_{H}^{*}$

(13) $$\begin{array}{c} E_{H}^{*}=\frac{\delta +\theta +\mu_{H}}{\varphi }I_{H}^{*},\;\;\\ Q_{H}^{*}=\frac{\theta }{\omega +\mu_{H}}\;\;I_{H}^{*},\\ R_{H}^{*}=\frac{\delta (\omega +\mu_{H})+\omega \theta }{\tau +\mu_{H}\left(\omega +\mu_{H}\right)}I_{H}^{*}. \end{array}$$

#### Analysis of sensitivity

$${ℑ}_{V}^{R_{0}}=\frac{\partial R_{0}}{\partial v}\times \frac{v}{\partial R_{0}}$$

Since,

$R_{0}=\left(1+\varepsilon \right)\frac{\Lambda_{H}}{\mu_{H}}\frac{\varphi }{\left(\varphi +\mu_{H}\right)\left(\delta +\theta +\mu_{H}\right)}.\frac{\rho \Lambda }{\mu_{R}}$.

At $v=\Lambda_{H},\;\;\;\;\;\;{ℑ}_{v}^{R_{0}}=\frac{\partial R_{0}}{\partial v}\times \frac{v}{\partial R_{0}}=1$

Similarly

(14) $$\begin{array}{c} {ℑ}_{v}^{R_{0}}={ℑ}_{\varepsilon }^{R_{0}}=1,\;\;\;\;\;\;\;\;{ℑ}_{v}^{R_{0}}={ℑ}_{\varphi }^{R_{0}}=1,\;\;\;\;\;\;\;\;\;\;\;{ℑ}_{v}^{R_{0}}={ℑ}_{\delta }^{R_{0}}=1,\;\;\;\;\;\;\;\;\;\;\;{ℑ}_{v}^{R_{0}}={ℑ}_{\theta }^{R_{0}}=1,\;\;\;\;\;\;\;\;\;\;{ℑ}_{v}^{R_{0}}={ℑ}_{\rho }^{R_{0}}=1,\\ {ℑ}_{v}^{R_{0}}={ℑ}_{\mu_{H}}^{R_{0}}=1,\;\;\;\;\;\;\;\;\;\;\;\;{ℑ}_{v}^{R_{0}}={ℑ}_{\Lambda }^{R_{0}}=1,\;\;\;\;\;\;\;\;\;\;\;\;{ℑ}_{v}^{R_{0}}={ℑ}_{\mu_{R}}^{R_{0}}=1.\;\;\;\;\;{X_{\tau }}^{R_{0}}={X_{\lambda }}^{R_{0}}=1 \end{array}$$

That basically indicates that the model is extremely sensitive to changes in a specific variable or parameter.

#### Global stability of the endemic equilibrium

Theorem 3.1 *Assume* $R_{0}>1$ *and that the endemic equilibrium*

(15) $$E^{*}=\left(S_{H}^{*},E_{H}^{*},I_{H}^{*},Q_{H}^{*},\;\;R_{H}^{*},S_{R}^{*},I_{R}^{*}.\right)$$

of system (2) exists and lies in invariant region Ω. Further assume all parameters are positive and that the incidence terms are continuously differentiable onΩ. Then $E^{*}$ globally asymptotically stable Ωin the sense of Mittag–Leffler stability at Atangana–Baleanu–Caputo (ABC) fractional dynamics

**Proof:**

Consider the Lyapunov function.

(16) $$\nu \left(t\right)=\sum_{x\in X}w_{x}\left(x\left(t\right)-x^{*}-x^{*}\ln\frac{x(t)}{x^{*}}\right),$$

Where ν(t) is positive definite with respect to the endemic equilibrium $E^{*}$ $X=(S_{H},E_{H},I_{H},Q_{H},\;\;R_{H},S_{R},I_{R}.)$denotes the corresponding endemic equilibrium component, and $w_{x}>0$ are constant weights chosen to balance terms (specified below).

v(t)≥0 ∀t≥0 and v(t)=0 $\Leftrightarrow x\left(t\right)=x^{*}$ for all x∈X .

Using the inequality (valid fory>0)

(17) $$\left(1-\frac{1}{y}\right)\left(y-1\right)\geq 0\;\;\;\Leftrightarrow \left(y-1\right)-\ln y\geq 0,$$

each term in the derivative contributes non-positively, and therefore ${}^{ABC}D{}_{t}^{\eta }\nu \left(t\right)\leq 0$:

Equality holds if and only if $S_{H}=S_{H}^{*},\;E_{H}=E_{H}^{*},\;I_{H}=I_{H}^{*},\;\;Q_{H}=Q_{H}^{*},\;\;R_{H}=R_{H}^{*},S_{R}=S_{R}^{*},I_{R}=I_{R}^{*}.$ each summand is nonnegative, hence V is positive definite with respect to

$E^{*}$For the ABC fractional derivative, we use the standard Lyapunov comparison argument employed for non-singular kernel fractional operators:

${}^{ABC}D{}_{t}^{\eta }\nu \left(t\right)\leq 0$ largest invariant set contained in.

{ ${}^{ABC}D{}_{t}^{\eta }\nu \left(t\right)=0$} is then globally asymptotically stable in Ω (LaSalle-type invariance principle for fractional systems).

Differentiate V along solutions. For each state x(t),

(18) $$\frac{d}{dt}\left(x-x^{*}-x^{*}\ln\frac{x}{x^{*}}\right)=\left(1-\frac{x^{*}}{x}\right)\overset{\circ \cdot }{x},$$

Consequently, along trajectories,

(19) $${}^{ABC}D{}_{t}^{\eta }\nu \left(t\right)=\sum_{x\in X}\left(1-\frac{x^{*}}{x}\right){}^{ABC}D{}_{t}^{\eta }x\left(t\right),$$

and we substitute the right-hand sides from the model equations (2).

Now choose weights $w_{x}$so that all recruitment and linear transfer terms cancel in pairs when grouped (e.g., terms moving from$E_{H}\to I_{H}$,$I_{H}\to Q_{H},$$Q_{H}\to R_{H}$, etc.).

This is standard in epidemic Lyapunov constructions: pick $wE_{H},wI_{H},wQ_{H},wR_{H}$ ​​ such that coefficients of the internal transition terms satisfy balance identities. With these cancellations, the remaining terms can be arranged into two groups: Strictly dissipative linear loss terms, each producing a non-positive contribution of the form

(20) $$-k_{z}w_{x}\frac{{\left(x-x^{*}\right)}^{2}}{x}\leq 0,k_{z}>0$$

which follows from

(21) $$\left(1-\frac{x^{*}}{x}\right)\left(x-x^{*}\right)=\frac{{\left(x-x^{*}\right)}^{2}}{x}\geq 0$$

Incidence (infection) terms, which can be written using the equilibrium identities at$E^{*}$ For example, any incidence term of the form $F(S_{H},I_{H},I_{R},x)$ satisfies at equilibrium $F(S_{H}^{*},I_{H}^{*},I_{R}^{*},x^{*})$corresponding outflow at $E^{*}$.

After substitution and simplification, these terms collect into expressions proportional to

(22) $$\psi \left(y\right)=\left(y-1\right)-\ln y\geq 0\;\text{with}\;y=\frac{x(t)}{x^{*}}$$

and therefore they contribute non-positively to ${}^{ABC}D^{\eta }v\left(t\right)$ once grouped appropriately.

(23) $$\text{Hence}\text{we}\text{obtain}{}^{ABC}D^{\eta }v\left(t\right)\leq 0,\;\;\forall \;\;t\geq 0$$

with equality holding if and only if every term of the form $\frac{{\left(x-x^{*}\right)}^{2}}{x}$ is zero and every

$\psi (\frac{x(t)}{x^{*}})$ is zero. Since ψ(y)=0 only wheny=1, the equality condition implies

(24) $$S_{H}=S_{H}^{*},E_{H}=E_{H}^{*},I_{H}=I_{H}^{*},Q_{H}=Q_{H}^{*},\;\;R_{H}=R_{H}^{*},S_{R}=S_{R}^{*},I_{R}=I_{R}^{*}$$

that is, the largest invariant set inside {${}^{ABC}D^{\eta }v\left(t\right)=0$} is the singleton {$E^{*}$}.

Consequently, the endemic equilibrium is globally asymptotically stable according to the fractional LaSalle invariance principle in Ω.

#### Existence and uniqueness analysis

The existence–uniqueness proof is presented using compact operator notation, where all kernel functions are assumed continuous and bounded on the solution domain. The existence of a solution is needed in the process of proving the well-posedness of the mathematical model. To show that solutions exist and are unique, the functional form of the system (2) is built and studied in the context of fixed-point theory.

(25) $$\begin{array}{c} {}_{0}^{ABC}D_{t}^{\eta }S_{H}\left(t\right)=G_{1}{\left(t,S_{H}\left(t\right)\right)}_{0}^{ABC},\;\;{\;}_{0}^{ABC}D_{t}^{\eta }E_{H}\left(t\right)=G_{2}{\left(t,E_{H}\left(t\right)\right)}_{0}^{ABC}{,}_{0}^{ABC}D_{t}^{\eta }I_{H}\left(t\right)=G_{3}{\left(t,I_{H}\left(t\right)\right)}_{0}^{ABC},\\ {}_{0}^{ABC}D_{t}^{\eta }Q_{H}\left(t\right)=G_{4}{\left(t,Q_{H}\left(t\right)\right)}_{0}^{ABC}{,}_{0}^{ABC}D_{t}^{\eta }R_{H}\left(t\right)=G_{5}{\left(t,R_{H}\left(t\right)\right)}_{0}^{ABC}{,}_{0}^{ABC}D_{t}^{\eta }S_{R}\left(t\right)=G_{6}{\left(t,S_{R}\left(t\right)\right)}_{0}^{ABC},\\ {}_{0}^{ABC}D_{t}^{\eta }I_{R}\left(t\right)=G_{7}{\left(t,I_{R}\left(t\right)\right)}_{0}^{ABC}. \end{array}\}$$

The integral operator can be used on both sides of (25), in accordance with definition 2, so that

(26) $$\begin{array}{c} S_{H}\left(t\right)-S_{H}\left(0\right)=\frac{2\left(1-b\right)}{\left(2-b\right)M\left(b\right)}G_{1}\left(t,S_{H}\left(t\right)\right)+\frac{2b}{\left(2-b\right)F\left(b\right)}\int_{0}^{t}G_{1}\left(b,S_{H}\right)db,\\ E_{H}\left(t\right)-E_{H}\left(0\right)=\frac{2\left(1-b\right)}{\left(2-b\right)M\left(b\right)}G_{2}\left(t,E_{H}\left(t\right)\right)+\frac{2b}{\left(2-b\right)F\left(b\right)}\int_{0}^{t}G_{2}\left(b,E_{H}\right)db,\\ I_{H}\left(t\right)-I_{H}\left(0\right)=\frac{2\left(1-b\right)}{\left(2-b\right)M\left(b\right)}G_{3}\left(t,I_{H}\left(t\right)\right)+\frac{2b}{\left(2-b\right)F\left(b\right)}\int_{0}^{t}G_{3}\left(b,I_{H}\right)db,\\ Q_{H}\left(t\right)-Q_{H}\left(0\right)=\frac{2\left(1-b\right)}{\left(2-b\right)M\left(b\right)}G_{4}\left(t,Q_{H}\left(t\right)\right)+\frac{2b}{\left(2-b\right)F\left(b\right)}\int_{0}^{t}G_{4}\left(b,Q_{H}\right)db,\\ R_{H}\left(t\right)-R_{H}\left(0\right)=\frac{2\left(1-b\right)}{\left(2-b\right)M\left(b\right)}G_{5}\left(t,R_{H}\left(t\right)\right)+\frac{2b}{\left(2-b\right)F\left(b\right)}\int_{0}^{t}G_{5}\left(b,R_{H}\right)db,\\ S_{R}\left(t\right)-S_{R}\left(0\right)=\frac{2\left(1-b\right)}{\left(2-b\right)M\left(b\right)}G_{6}\left(t,S_{R}\left(t\right)\right)+\frac{2b}{\left(2-b\right)F\left(b\right)}\int_{0}^{t}G_{6}\left(b,S_{R}\right)db,\\ I_{R}\left(t\right)-I_{R}\left(0\right)=\frac{2\left(1-b\right)}{\left(2-b\right)M\left(b\right)}G_{7}\left(t,I_{R}\left(t\right)\right)+\frac{2b}{\left(2-b\right)F\left(b\right)}\int_{0}^{t}G_{7}\left(b,I_{R}\right)db, \end{array}$$

Compact operator paragraph above, following the Lipschitz condition.

**Theorem**

The Lipchitz criterion of contraction is satisfied by kernel $G_{1}$ this inequality hold;

$$0\leq \left(\left(1-\in \right){\left(\gamma +a\right)}^{b}∥I_{H}+I_{R}∥+{\mu_{H}}^{b}+\tau^{b}\right)<1$$

**Proof**

(27) $$\begin{array}{c} ∥G_{1}\left(t,S_{H}\right)-G_{1}\left(t,{S_{H}}_{1}\right)∥=∥\begin{array}{c} \left(1-\in \right){\left(\gamma +a\right)}^{b}I\left[S_{H}\left(t\right)-{S_{H}}_{1}\left(t\right)\right]-\\ \left[{\mu_{H}}^{b}+\tau^{b}\right]\left[S_{H}\left(t\right)-S_{H}\leq ∥I∥\;{∥S_{H}\left(t\right)-{S_{H}}_{1}\left(t\right)∥}_{1}\left(t\right)\right] \end{array}∥\\ +∥{\mu_{H}}^{b}+\tau^{b}∥∥S_{H}\left(t\right)-{S_{H}}_{1}\left(t\right)∥\leq ∥S_{H}\left(t\right)-{S_{H}}_{1}\left(t\right)∥\;∥S_{H}\left(t\right)-{S_{H}}_{1}\left(t\right)∥∥\left(1-\in \right){\left(\gamma +\alpha \right)}^{b}∥+\left({\mu_{H}}^{b}+\tau^{b}\right)∥. \end{array}$$

Let$\nabla_{1}=\left(1-\in \right){\left(\gamma +\alpha \right)}^{b}∥I∥+\left({\mu_{H}}^{b}+\tau^{b}\right)∥$, and$∥I∥\leq \ell_{1}$, is bounded such that

(28) $$∥G_{1}\left(t,S_{H}\right)-G_{1}\left(t,{S_{H}}_{1}\right)∥\leq \nabla_{1}∥S_{H}\left(t\right)-{S_{H}}_{1}\left(t\right)∥.$$

Lipchitz condition is satisfied for$\nabla_{1}$, $0\leq \left(1-\in \right){\left(\gamma +\alpha \right)}^{b}∥I∥+\left({\mu_{H}}^{b}+\tau^{b}\right)∥<1$and

$\nabla_{1}$Contracts. Also Lipchitz condition of other functional as follow.

$$∥G_{2}\left(t,E_{H}\right)-G_{2}\left(t,E_{H}\right)∥\leq \nabla_{2}∥E_{H}\left(t\right)-E_{H}\left(t\right)∥,\;∥G_{3}\left(t,I_{H}\right)-G_{3}\left(t,I_{H}\right)∥\leq \nabla_{3}∥I_{H}\left(t\right)-I_{H}\left(t\right)∥$$

$$∥G_{4}\left(t,Q\right)-G_{4}\left(t,Q\right)∥\leq \nabla_{4}∥Q\left(t\right)-Q\left(t\right)∥,\;∥G_{5}\left(t,R_{H}\right)-G_{5}\left(t,R_{H}\right)∥\leq G_{5}∥R_{H}\left(t\right)-R_{H}\left(t\right)∥$$

(29) $$∥G_{6}\left(t,S_{R}\right)-G_{6}\left(t,S_{R}\right)∥\leq \nabla_{6}∥S_{R}\left(t\right)-S_{R}\left(t\right)∥,\;∥G_{7}\left(t,I_{R}\right)-G_{7}\left(t,I_{R}\right)∥\leq \nabla_{7}∥I_{H}\left(t\right)-I_{H}\left(t\right)∥.$$

And a contraction occurs for each $\nabla_{2}=0\leq {\left(\varphi +\mu_{H}\right)}^{b}<1$, $\nabla_{3}=0\leq {\left(\delta +\theta +\mu_{H}\right)}^{b}<1$, $\nabla_{4}=0\leq {\left(\delta +\theta +\mu_{H}\right)}^{b}<1$,$\nabla_{5}=0\leq {\left(\omega +\mu_{Q}\right)}^{b}<1$,$\nabla_{6}=0\leq {\mu^{b}}_{R}<1$,$\nabla_{7}=0\leq {\mu^{b}}_{R}<1$.

Furthermore following recursive form given by:

(30) $$\begin{array}{c} {ℑ}_{1n}\left(t\right)={S_{H}}_{n}\left(t\right)-{S_{H}}_{n-1}\left(t\right)\frac{2\left(1-b\right)}{bM\left(b\right)}\left[G_{1}\left(t,{S_{H}}_{n-1}\right)-G_{1}\left(t,{S_{H}}_{n-2}\right)\right]+\frac{2b}{\left(2-b\right)M\left(b\right)}\int_{0}^{t}G_{1}\left(b,{S_{H}}_{n-1}\right)db,\\ {ℑ}_{2n}\left(t\right)={E_{H}}_{n}\left(t\right)-{E_{H}}_{n-1}\left(t\right)\frac{2\left(1-b\right)}{bM\left(b\right)}\left[G_{2}\left(t,{E_{H}}_{n-1}\right)-G_{2}\left(t,{E_{H}}_{n-2}\right)\right]+\frac{2b}{\left(2-b\right)M\left(b\right)}\int_{0}^{t}G_{2}\left(b,{E_{H}}_{n-1}\right)db,\\ {ℑ}_{3n}\left(t\right)={I_{H}}_{n}\left(t\right)-{I_{H}}_{n-1}\left(t\right)\frac{2\left(1-b\right)}{bM\left(b\right)}\left[G_{3}\left(t,{I_{H}}_{n-1}\right)-G_{3}\left(t,{I_{H}}_{n-2}\right)\right]+\frac{2b}{\left(2-b\right)M\left(b\right)}\int_{0}^{t}G_{3}\left(b,{I_{H}}_{n-1}\right)db,\\ {ℑ}_{4n}\left(t\right)={Q_{H}}_{n}\left(t\right)-{Q_{H}}_{n-1}\left(t\right)\frac{2\left(1-b\right)}{bM\left(b\right)}\left[G_{4}\left(t,{Q_{H}}_{n-1}\right)-G_{4}\left(t,{Q_{H}}_{n-2}\right)\right]+\frac{2b}{\left(2-b\right)M\left(b\right)}\int_{0}^{t}G_{4}\left(b,{Q_{H}}_{n-1}\right)db, \end{array}$$

$$\begin{array}{c} {ℑ}_{5n}\left(t\right)={R_{H}}_{n}\left(t\right)-{R_{H}}_{n-1}\left(t\right)\frac{2\left(1-b\right)}{bM\left(b\right)}\left[G_{5}\left(t,{R_{H}}_{n-1}\right)-G_{4}\left(t,{R_{H}}_{n-2}\right)\right]+\frac{2b}{\left(2-b\right)M\left(b\right)}\int_{0}^{t}G_{5}\left(b,{R_{H}}_{n-1}\right)db,\\ {ℑ}_{6n}\left(t\right)={S_{R}}_{n}\left(t\right)-{S_{R}}_{n-1}\left(t\right)\frac{2\left(1-b\right)}{bM\left(b\right)}\left[G_{6}\left(t,{S_{R}}_{n-1}\right)-G_{6}\left(t,{S_{R}}_{n-2}\right)\right]+\frac{2b}{\left(2-b\right)M\left(b\right)}\int_{0}^{t}G_{6}\left(b,{S_{R}}_{n-1}\right)db,\\ {ℑ}_{7n}\left(t\right)={I_{R}}_{n}\left(t\right)-{I_{R}}_{n-1}\left(t\right)\frac{2\left(1-b\right)}{bM\left(b\right)}\left[G_{7}\left(t,{I_{R}}_{n-1}\right)-G_{7}\left(t,{I_{R}}_{n-2}\right)\right]+\frac{2b}{\left(2-b\right)M\left(b\right)}\int_{0}^{t}G_{7}\left(b,{I_{R}}_{n-1}\right)db. \end{array}$$

Where b denotes the integration variable, and M(b),F(b),U(b) are bounded kernel functions associated with the ABC fractional integral operator.

Norm of the above system to obtain.

$$∥{ℑ}_{1n}\left(t\right)∥=∥{S_{H}}_{n}\left(t\right)-{S_{H}}_{n-1}\left(t\right)∥=∥\frac{2\left(1-b\right)}{\left(2-b\right)M\left(b\right)}\left[G_{1}\left(t,{S_{H}}_{n-1}\right)-G_{1}\left(t_{1},{S_{H}}_{n-2}\right)\right]+\frac{2b}{\left(2-b\right)Y\left(b\right)}\int_{0}^{t}G_{1}\left(b,{S_{H}}_{n-1}\right)-G_{1}\left(t_{1},{S_{H}}_{n-2}\right)db∥,$$

(31) $$\Rightarrow ∥{ℑ}_{1n}\left(t\right)∥\leq \frac{2\left(1-b\right)}{\left(2-b\right)M\left(b\right)}∥\left[G_{1}\left(t,{S_{H}}_{n-1}\right)-G_{1}\left(t_{1},{S_{H}}_{n-2}\right)\right]∥+\frac{2b}{\left(2-b\right)Mb}∥\int_{0}^{t}U_{1}\left(b,{S_{H}}_{n-1}\right)-U_{1}\left(t_{1},{S_{H}}_{n-2}\right)db∥.$$

Following the Lipchitz condition;

$$∥{ℑ}_{1n}\left(t\right)∥\leq \frac{2\left(1-b\right)}{\left(2-b\right)M\left(b\right)}\nabla_{1}∥{ℑ}_{1\left(n-1\right)}\left(t\right)∥+\frac{2b}{\left(2-b\right)M\left(b\right)}\nabla_{1}\times \int_{0}^{t}∥{ℑ}_{1\left(n-1\right)}\left(b\right)∥db,$$

$$∥{ℑ}_{2n}\left(t\right)∥\leq \frac{2\left(1-b\right)}{\left(2-b\right)M\left(b\right)}\nabla_{2}∥{ℑ}_{2\left(n-1\right)}\left(t\right)∥+\frac{2b}{\left(2-b\right)M\left(b\right)}\nabla_{2}\times \int_{0}^{t}∥{ℑ}_{2\left(n-1\right)}\left(b\right)∥db.$$

(32) $$\text{In}\text{general},\;∥{ℑ}_{in}\left(t\right)∥\leq \frac{2\left(1-b\right)}{\left(2-b\right)M\left(b\right)}\nabla_{i}∥{ℑ}_{i\left(n-1\right)}\left(t\right)∥+\frac{2b}{\left(2-b\right)M\left(b\right)}\nabla_{i}\times \int_{0}^{t}∥{ℑ}_{i\left(n-1\right)}\left(b\right)∥db,\;\;\;\;\;\;i=3..8$$

Therefore, by the Banach Fixed-Point Theorem, system (2) admits a unique continuous solution on the interval [0,T].

### Experiments with LADM for model solving

This research adopts the Laplace Adomian Decomposition Method (LADM) as it is especially effective with nonlinear fractional differential equations based on the use of Atangana Baleanu Caputo operators. Compared to traditional finite-difference methods, LADM eliminates the linearization and discretization errors, maintains the nonlocal memory structure of the ABC derivatives, and offers fast convergent semi-analytical approximations to nonlinear epidemic models. By applying the LT both sides of model (2), we can obtain the general method of the model (2) with initial condition**.**

(33) $$\begin{array}{c} L\left[{}^{ABC}D{}_{t}^{\eta }S_{H}\left(t\right)\right]=L\left[\Lambda_{H}-S_{H}\left(1-\in \right)\left(\gamma I_{H}+aI_{R}\right)-\mu_{H}S_{H}+\tau R_{H}\right],\\ L\left[{}^{ABC}D{}_{t}^{\eta }E_{H}\left(t\right)\right]=L\left[S_{H}\left(1-\in \right)\left(\gamma I_{H}+aI_{R}\right)-\left(\varphi +\mu_{H}\right)E_{H}\right],\\ L\left[{}^{ABC}D{}_{t}^{\eta }I_{H}\left(t\right)\right]=L\left[\varphi {E^{\prime }}_{H}\left(t\right)-\left(\delta +\theta +\mu_{H}\right)I_{H}\right],\\ L\left[{}^{ABC}D{}_{t}^{\eta }Q_{H}\left(t\right)\right]=L\left[\theta I_{H}-\left(\omega +\mu_{Q}\right)Q_{H}\right],\\ L\left[{}^{ABC}D{}_{t}^{\eta }R_{H}\left(t\right)\right]=L\left[\delta I_{H}-\omega Q_{H}-\left(\tau +\mu_{H}\right)R_{H}\right],\\ L\left[{}^{ABC}D{}_{t}^{\eta }S_{R}\left(t\right)\right]=L\left[\Lambda_{R}-\rho S_{R}I_{R}-\mu_{R}S_{R}\right],\\ L\left[{}^{ABC}D{}_{t}^{\eta }I_{R}\left(t\right)\right]=L\left[\rho S_{R}I_{R}-\mu_{R}I_{R}\right]. \end{array}$$

Eq. (33) implies

(34) $$\begin{array}{c} s^{\eta }S_{H}\left(t\right)-s^{\eta -1}S_{H}\left(0\right)=L\left[\Lambda_{H}-S_{H}\left(1-\in \right)\left(\gamma I_{H}+aI_{R}\right)-\mu_{H}S_{H}+\tau R_{H}\right],\\ s^{\eta }E_{H}\left(t\right)-s^{\eta -1}E_{H}\left(0\right)=L\left[S_{H}\left(1-\in \right)\left(\gamma I_{H}+aI_{R}\right)-\left(\varphi +\mu_{H}\right)E_{H}\right],\\ s^{\eta }I_{H}\left(t\right)-s^{\eta -1}I_{H}\left(0\right)=L\left[\varphi {E^{\prime }}_{H}\left(t\right)-\left(\delta +\theta +\mu_{H}\right)I_{H}\right],\\ s^{\eta }Q_{H}\left(t\right)-s^{\eta -1}Q_{H}\left(0\right)=L\left[\theta I_{H}-\left(\omega +\mu_{Q}\right)Q_{H}\right],\\ s^{\eta }R_{H}\left(t\right)-s^{\eta -1}R_{H}\left(0\right)=L\left[\delta I_{H}-\omega Q_{H}-\left(\tau +\mu_{H}\right)R_{H}\right]\\ s^{\eta }S_{R}\left(t\right)-s^{\eta -1}S_{R}\left(0\right)=L\left[\Lambda_{R}-\rho S_{R}I_{R}-\mu_{R}S_{R}\right],\\ s^{\eta }I_{R}\left(t\right)-s^{\eta -1}I_{R}\left(0\right)=L\left[\rho S_{R}I_{R}-\mu_{R}I_{R}.\right] \end{array}$$

From Eq. (34)

(35) $$\begin{array}{c} s^{\eta }S_{H}\left(t\right)=s^{\eta -1}S_{H}\left(0\right)+L\left[\Lambda_{H}-S_{H}\left(1-\in \right)\left(\gamma I_{H}+aI_{R}\right)-\mu_{H}S_{H}+\tau R_{H}\right],\\ s^{\eta }E_{H}\left(t\right)=s^{\eta -1}E_{H}\left(0\right)+L\left[S_{H}\left(1-\in \right)\left(\gamma I_{H}+aI_{R}\right)-\left(\varphi +\mu_{H}\right)E_{H}\right],\\ s^{\eta }I_{H}\left(t\right)=s^{\eta -1}I_{H}\left(0\right)+L\left[\varphi {E^{\prime }}_{H}\left(t\right)-\left(\delta +\theta +\mu_{H}\right)I_{H}\right],\\ s^{\eta }Q_{H}\left(t\right)=s^{\eta -1}Q_{H}\left(0\right)+L\left[\theta I_{H}-\left(\omega +\mu_{Q}\right)Q_{H}\right],\\ s^{\eta }R_{H}\left(t\right)=s^{\eta -1}R_{H}\left(0\right)+L\left[\delta I_{H}-\omega Q_{H}-\left(\tau +\mu_{H}\right)R_{H}\right]\\ s^{\eta }S_{R}\left(t\right)=s^{\eta -1}S_{R}\left(0\right)+L\left[\Lambda_{R}-\rho S_{R}I_{R}-\mu_{R}S_{R}\right],\\ s^{\eta }I_{R}\left(t\right)=s^{\eta -1}I_{R}\left(0\right)+L\left[\rho S_{R}I_{R}-\mu_{R}I_{R}.\right] \end{array}$$

By solving Eq. (35), we have Eq. (36)

(36) $$\begin{array}{c} S_{H}\left(t\right)=s^{-1}S_{H}\left(0\right)+\frac{1}{s^{\eta }}L\left[\Lambda_{H}-S_{H}\left(1-\in \right)\left(\gamma I_{H}+aI_{R}\right)-\mu_{H}S_{H}+\tau R_{H}\right],\\ E_{H}\left(t\right)=s^{-1}E_{H}\left(0\right)+\frac{1}{s^{\eta }}L\left[S_{H}\left(1-\in \right)\left(\gamma I_{H}+aI_{R}\right)-\left(\varphi +\mu_{H}\right)E_{H}\right],\\ I_{H}\left(t\right)=s^{-1}I_{H}\left(0\right)+\frac{1}{s^{\eta }}L\left[\varphi {E^{\prime }}_{H}\left(t\right)-\left(\delta +\theta +\mu_{H}\right)I_{H}\right],\\ Q_{H}\left(t\right)=s^{-1}Q_{H}\left(0\right)+\frac{1}{s^{\eta }}L\left[\theta I_{H}-\left(\omega +\mu_{Q}\right)Q_{H}\right],\\ R_{H}\left(t\right)=s^{-1}R_{H}\left(0\right)+\frac{1}{s^{\eta }}L\left[\delta I_{H}-\omega Q_{H}-\left(\tau +\mu_{H}\right)R_{H}\right],\\ S_{R}\left(t\right)=s^{-1}S_{R}\left(0\right)+\frac{1}{s^{\eta }}L\left[\Lambda_{R}-\rho S_{R}I_{R}-\mu_{R}S_{R}\right],\\ I_{R}\left(t\right)=s^{\eta -1}I_{R}\left(0\right)+\frac{1}{s^{\eta }}L\left[\rho S_{R}I_{R}-\mu_{R}I_{R}\right]. \end{array}$$

$S_{H}\left(t\right),E_{H}\left(t\right),I_{H}\left(t\right),Q_{H}\left(t\right),R_{H}\left(t\right),S_{R}\left(t\right),I_{R}\left(t\right).$ are in form infinite series by

(37) $$\begin{array}{c} S_{H}\left(t\right)=\sum_{n=0}^{\propto }{S_{H}}_{n},.E_{H}\left(t\right)=\sum_{n=0}^{\propto }{E_{H}}_{n},I_{H}\left(t\right)=\sum_{n=0}^{\propto }{I_{H}}_{n},Q_{H}\left(t\right)=\sum_{n=0}^{\propto }Q_{Hn},.R_{H}\left(t\right)=\sum_{n=0}^{\propto }{R_{H}}_{n},S_{R}\left(t\right)=\sum_{n=0}^{\propto }{S_{R}}_{n},\\ I_{R}\left(t\right)=\sum_{n=0}^{\propto }I_{Rn}, \end{array}$$

Putting (37) in (36) Using initial condition in equation, and to obtain (38)l

(38) $$\begin{array}{c} \sum_{n=0}^{\infty }S_{Hn}\left(t\right)=L^{-1}\left[\frac{1}{s^{\eta }}L\left[\Lambda_{H}-S_{H}\left(1-\in \right)\left(\gamma I_{H}+aI_{R}\right)-\mu_{H}S_{H}+\tau R_{H}\right]\right],\\ \sum_{n=0}^{\infty }E_{Hn}\left(t\right)=L^{-1}\left[\frac{1}{s^{\eta }}L\left[S_{H}\left(1-\in \right)\left(\gamma I_{H}+aI_{R}\right)-\left(\varphi +\mu_{H}\right)E_{H}\right]\right],\\ \sum_{n=0}^{\infty }I_{H}\left(t\right)=L^{-1}\left[\frac{1}{s^{\eta }}L\left[\varphi {E^{\prime }}_{H}\left(t\right)-\left(\delta +\theta +\mu_{H}\right)I_{H}\right]\right],\\ \sum_{n=0}^{\infty }Q_{H}\left(t\right)=L^{-1}\left[\frac{1}{s^{\eta }}L\left[\theta I_{H}-\left(\omega +\mu_{Q}\right)Q_{H}\right]\right],\\ \sum_{n=0}^{\infty }R_{H}\left(t\right)=L^{-1}\left[\frac{1}{s^{\eta }}L\left[\delta I_{H}-\omega Q_{H}-\left(\tau +\mu_{H}\right)R_{H}\right]\right],\\ \sum_{n=0}^{\infty }S_{R}\left(t\right)=L^{-1}\left[\frac{1}{s^{\eta }}L\left[\Lambda_{R}-\rho S_{R}I_{R}-\mu_{R}S_{R}\right]\right],\\ \sum_{n=0}^{\infty }I_{R}\left(t\right)=L^{-1}\left[\frac{1}{s^{\eta }}L\left[\rho S_{R}I_{R}-\mu_{R}I_{R}\right]\right]. \end{array}$$

The approximate solution is expected to be reached when the limit for n gets closer to infinity.

$$\begin{array}{c} S_{h}\left(t\right)=\lim_{y\to \infty }S_{hy}\left(t\right),\;E_{h}\left(t\right)=\lim_{y\to \infty }E_{hy}\left(t\right),\;\;I_{h}\left(t\right)=\lim_{y\to \infty }I_{hy}\left(t\right),\;\;\;Q_{h}\left(t\right)=\lim_{y\to \infty }Q_{hy}\left(t\right),\;V_{h}\left(t\right)=\lim_{y\to \infty }V_{hy}\left(t\right),\;\\ \;R_{h}\left(t\right)=\lim_{y\to \infty }R_{hy}\left(t\right),S_{r}\left(t\right)=\lim_{y\to \infty }S_{ry}\left(t\right),\;E_{r}\left(t\right)=\lim_{y\to \infty }E_{ry}\left(t\right),\;\;I_{r}\left(t\right)=\lim_{y\to \infty }I_{ry}\left(t\right),E_{c}\left(t\right)=\lim_{y\to \infty }E_{cy}\left(t\right). \end{array}$$

## Optimal control formulation and analysis

Methods section gives qualitative analyses which conducted on the uncontrolled epidemic system. We now generalize the model to time-dependent control variables.$x_{1}\left(t\right)$ and$x_{2}\left(t\right)$) to formulate the optimal control problem.

An optimal control problem for the fractional-order Lassa fever model is formulated and examined in this section. Public enlightenment and quarantine enforcement are treated as decision variables whose optimal deployment minimizes disease burden while accounting for implementation costs. The formulation aligns with decision-oriented epidemic control

(39) $$\begin{array}{c} {}^{ABC}D{}_{t}^{\eta }S_{H}\left(t\right)=\Lambda_{H}-\left(1-x_{1}\left(t\right)\right)S_{H}\left(1-\in \right)\left(\gamma I_{H}+aI_{R}\right)-\mu_{H}S_{H}+\tau R_{H},\\ {}^{ABC}D{}_{t}^{\eta }E_{H}\left(t\right)=\left(1-x_{1}\left(t\right)\right)S_{H}\left(1-\in \right)\left(\gamma I_{H}+aI_{R}\right)-\left(\varphi +\mu_{H}\right)E_{H},\\ {}^{ABC}D{}_{t}^{\eta }I_{H}\left(t\right)=\varphi {E^{\prime }}_{H}\left(t\right)-\left(\delta +\theta +\mu_{H}+x_{2}\left(t\right)\right)I_{H},\\ {}^{ABC}D{}_{t}^{\eta }Q_{H}\left(t\right)=x_{2}\left(t\right)\theta I_{H}-\left(\omega +\mu_{Q}\right)Q_{H},\\ {}^{ABC}D{}_{t}^{\eta }R_{H}\left(t\right)=\delta I_{H}-\omega Q_{H}-\left(\tau +\mu_{H}\right)R_{H,}\\ {}^{ABC}D{}_{t}^{\eta }S_{R}\left(t\right)=\Lambda_{R}-\rho S_{R}I_{R}-\mu_{R}S_{R},\\ {}^{ABC}D{}_{t}^{\eta }I_{R}\left(t\right)=\rho S_{R}I_{R}-\mu_{R}I_{R}. \end{array}$$

### Controlled system

Let $x_{1}\left(t\right)$denote the intensity of public enlightenment campaigns, which reduce effective transmission through behavioral modification. Infection reduction terms: $\left(1-x_{1}\left(t\right)\right)\left(1-\in \right)\left(\gamma I_{H}+aI_{R}\right)S_{H}$

$x_{2}\left(t\right)$denote the intensity of quarantine enforcement**,** which removes infectious individuals from active transmission. Quarantine transfer $x_{2}\left(t\right)\theta I_{H}$

Both controls are assumed to be measurable, bounded, and time-dependent, satisfying

(40) $$\begin{array}{c} X=\left(x_{1},x_{2}\right)\in L^{\infty }\left(0,T\right]\;\;:\\ 0\leq x_{1}\left(t\right)\leq {x_{1}}^{\max},\\ 0\leq x_{2}\left(t\right)\leq {x_{2}}^{\max} \end{array}$$

In (ABC) concept, the controlled fractional-order system is expressed by combining $x_{1}\left(t\right)$ into the transmission terms and $x_{2}\left(t\right)$ into the progression of infectious individuals to quarantine.

### Objective functional

The goal is to balance the operational and financial expenses of intervention deployment over a limited time horizon while minimizing the cumulative infectious burden.[0,T] The performance index is defined as

(41) $$J\left(x_{1},x_{2}\right)=\int_{0}^{T}\left[AI_{H}\left(t\right)+BQ_{H}\left(t\right)+\frac{1}{2}\left(C_{1}x_{1}^{2}\left(t\right)+C_{2}x_{1}^{2}\left(t\right)\right)\right]dt$$

where:

$I_{H}\left(t\right)$ is the infectious class, $Q_{H}\left(t\right)$ quarantined population,

A, B > 0 are weighting parameters reflecting public health priorities, C1​, C2​ >0 represent the relative costs of awareness and quarantine interventions.

The quadratic structure penalizes excessive control intensity while ensuring convexity of the optimization problem.

### An optimal control's existence

An ideal control pair's existence $(x_{1}^{*},x_{2}^{*})=X$follows from standard arguments in optimal Modified control theory for fractional-order systems: Convex, closed, and nonempty is the control set. Any admissible control has a unique solution in the bounded state system. The objective functional's integrand is convex in the controls. Below, the objective functional is bounded.

Therefore, there is at least one pair of optimal controls that minimizes$J(x_{1},x_{2})$.

### Pontryagin’s maximum principle

Pontryagin's Maximum Principle for fractional-order systems is used to describe the ideal controls. The definition of the Hamiltonian is

(42) $$H=AI_{H}\left(t\right)+BQ_{H}\left(t\right)+\frac{1}{2}\left(C_{1}x_{1}^{2}\left(t\right)+C_{2}x_{1}^{2}\left(t\right)\right)+\sum_{i=1}^{7}\lambda_{i}\left(t\right)f_{i}\left(y,x\right),$$

x(t)Vector state variables, $\lambda_{i}$ are the adjoint variables,$f_{i}$controlled state equations.

The adjoint equations are given by

(43) $${}^{ABC}D_{t}^{\alpha }\lambda_{i}\left(t\right)=\frac{\partial H}{\partial x_{i}},\lambda_{i}\left(T\right)=0,$$

for all state variables $x_{i}$.

### Adjoint system and necessary optimality conditions

The necessary conditions for optimality are obtained from Pontryagin’s Maximum Principle

${}^{ABC}D_{t}^{\alpha }\lambda_{i}\left(t\right)=-\frac{\partial H}{\partial X}\;\;\;\;$Where $\lambda_{i}\left(t\right)={\left(\lambda_{1},\lambda_{2},\lambda_{3},\lambda_{4},\lambda_{5},\lambda_{6},\lambda_{7}\right)}^{T}$is the adjoint vector corresponding to the state vector$X={\left(S_{H},\;E_{H},\;\;I_{H},\;\;Q_{H},\;R_{H},\;S_{R},\;I_{R}\right)}^{T}$

The corresponding transversely conditions are: $\lambda_{i}\left(T\right)=0,\;\;\;\;\;i=1,......,7$

Define the infection incidence term as $\Psi \left(t\right)=\left(1-x_{1}\left(t\right)\right)\left(1-\in \right)\left(\gamma I_{H}+aI_{R}\right)$

Then the Hamiltonian becomes.

(44) $$\begin{array}{c} H=AI_{H}\left(t\right)+BQ_{H}\left(t\right)+\frac{1}{2}\left(C_{1}x_{1}^{2}\left(t\right)+C_{2}x_{1}^{2}\left(t\right)\right)\\ +\lambda_{1}\left[\Lambda_{H}-\Psi S_{H}-\mu_{H}S_{H}+\tau R_{H}\right]\\ +\lambda_{2}\left[\Psi S_{H}-\left(\varphi +\mu_{H}\right)E_{H}\right]\\ +\lambda_{3}\left[\varphi {E^{\prime }}_{H}\left(t\right)-\left(\delta +\theta +\mu_{H}+x_{2}\left(t\right)\right)I_{H}\right]\\ +\lambda_{4}\left[x_{2}\left(t\right)\theta I_{H}-\left(\omega +\mu_{Q}\right)Q_{H}\right]\\ +\lambda_{5}\left[\delta I_{H}-\omega Q_{H}-\left(\tau +\mu_{H}\right)R_{H}\right]\\ +\lambda_{6}\left[\Lambda_{R}-\rho S_{R}I_{R}-\mu_{R}S_{R}\right]\\ +\lambda_{7}\left[\rho S_{R}I_{R}-\mu_{R}I_{R}\right] \end{array}$$

Thus, the adjoint system is

(45) $$\begin{array}{c} {}^{ABC}D_{t}^{\alpha }\lambda_{1}=\lambda_{1}\left(\Psi +\mu_{H}+\tau \right)-\lambda_{2}\Psi ,\\ {}^{ABC}D_{t}^{\alpha }\lambda_{2}=-\lambda_{3}\varphi +\lambda_{2}\left(\varphi +\mu_{H}\right),\\ {}^{ABC}D_{t}^{\alpha }\lambda_{3}=-A+\lambda_{3}(\delta +\theta +\mu_{H}+x_{2}-\lambda_{4}x_{2}-\lambda_{5}\delta \\ -\lambda_{2}\left(1-x_{1}\right)\left(1-\in \right)aS_{H}+\lambda_{1}\left(1-x_{1}\right)\left(1-\in \right)aS_{H},\\ {}^{ABC}D_{t}^{\alpha }\lambda_{4}=-B+\lambda_{4}\left(\omega +\mu_{H}\right)-\lambda_{5}\omega ,\\ {}^{ABC}D_{t}^{\alpha }\lambda_{5}=\mu_{H}\lambda_{5}-\tau \lambda_{1},\\ {}^{ABC}D_{t}^{\alpha }\lambda_{6}=\left(\rho I_{R}-\mu_{R}\right)-\lambda_{7}\rho I_{R},\\ {}^{ABC}D_{t}^{\alpha }\lambda_{7}=\mu_{R}\lambda_{7}-\lambda_{2}\left(1-x_{1}\right)\left(1-\in \right)\gamma S_{H}+\lambda_{1}\left(1-x_{1}\right)\left(1-\in \right)\gamma S_{H}. \end{array}$$

Characterization of optimal controls.

(46) $$\begin{array}{c} \frac{\partial H}{\partial x_{i}}=\Phi_{1}\left(t\right),\;\;\;\;\;\;\;\;\;\;\;\;\;\frac{\partial H}{\partial x_{2}}=\Phi_{2}\left(t\right),\\ \Phi_{1}\left(t\right)=C_{1}x_{1}\left(t\right)+\left(\lambda_{7}-\lambda_{2}\right)\left(1-\in \right)\left(\gamma I_{H}+aI_{R}\right)S_{H},\\ \Phi_{2}\left(t\right)=C_{2}x_{2}\left(t\right)+\left(\lambda_{4}-\lambda_{3}\right)I_{H} \end{array}$$

Where $\Phi_{1}\left(t\right)$ and $\Phi_{2}\left(t\right)$are functions of the state and adjoint variables representing the marginal benefit of control.

Thus, the optimal controls satisfy the projection formulas:

$$\Phi_{1}\left(t\right)=0,\;\;\;\;\;\Phi_{2}\left(t\right)=0$$

Hence

(47) $$\begin{array}{c} x_{1}^{*}\left(t\right)=\min\left\{x_{1}^{\max},\max\left(0,\frac{\Phi_{1}\left(t\right)}{C_{1}}\right)\right\},\\ x_{2}^{*}\left(t\right)=\min\left\{x_{2}^{\max},\max\left(0,\frac{\Phi_{2}\left(t\right)}{C_{2}}\right)\right\}. \end{array}$$

These control expressions define the optimal public enlightenment and quarantine strategies as bounded feedback controls, determined jointly by the epidemic state variables and the associated adjoint dynamics.

## Confirmation of the estimated parameter values using real-world data

A well-known statistical procedure of modeling and estimation is used in which systematic procedures like calibration, simulation, and comparison with actual data are involved. The steps make use of a nonlinear least square fitted curve in minimizing differences and increasing the accuracy of a model. Our study's objective is to examine the dynamics of Lassa fever transmission in Nigeria based on public awareness and daily data from the Nigeria Center for Disease Control report. The process can begin with gathering conventional Lassa data from the literature, fitting the model to the data, and then using Mean to assess the model's performance.

Absolute Percentage Error (MAPE). $MAPE=\frac{1}{N}\sum_{m=1}^{N}|\frac{j(v)-k(v)}{k(v)}|\times 100\%$

j(v) as well ask(v) show data derived by the infected variable using the real Lassa data of the infected person and the solution found from the differential equation using LADM, respectively; N is the number of data points. are the parameters that were fitted.

In order to ensure that this model prediction is reliable and accurate, this section verifies the estimated parameter values. A workable explanation of Lassa fever transmission depends on accurate parameter estimation. The parameterization was based on epidemiological data from Nigeria, but in certain cases, logical assumptions were made in the absence of direct data. The parameters used, along with an explanation of each and an approximation or source, are shown in Table 1.

The model was calibrated using reported Nigeria Lassa fever surveillance data. Parameter fitting was performed by minimizing prediction error between observed and simulated infectious cases. The calibration yielded:

MAPE=X% RMSE=Y indicating satisfactory agreement between model predictions and observed outbreak data. Due to limited data resolution, MAPE was used as the primary calibration accuracy measure.

In cases where parameter ranges were reported in literature, midpoint or fitted representative values were used to simulate after calibration against observed data of the Nigeria outbreaks.

## Numerical results

The dynamics of the fractional-order Lassa fever model and the influence of memory, enlightenment of the population, and quarantine are quantified by numerical experiments. Model solutions are obtained through the Laplace-Adomian Decomposition Method, and the initial conditions and the values of the parameters are provided in Table 1. In order to assess the impact of memory on the development of epidemics, a number of different fractional orders are considered.

### Fractional memory effects

**Fig. 1:**
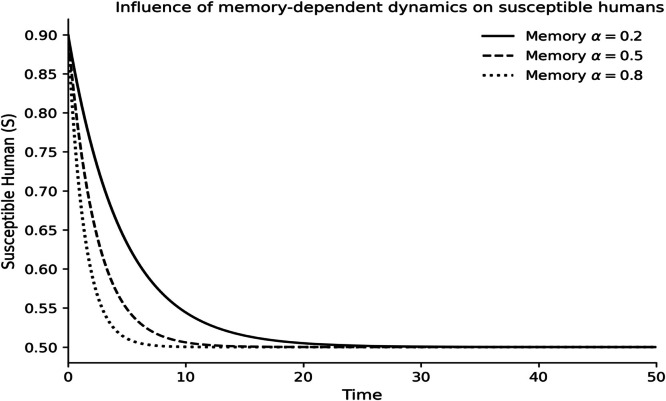
Influence of the memory-dependent dynamics on susceptible human.

**Fig. 2:**
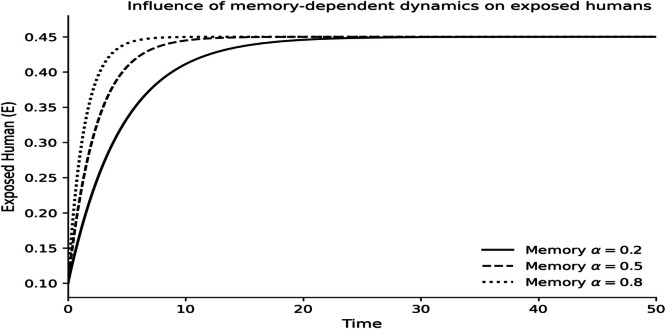
Influence of the memory-dependent dynamics on exposed human.

**Fig. 3:**
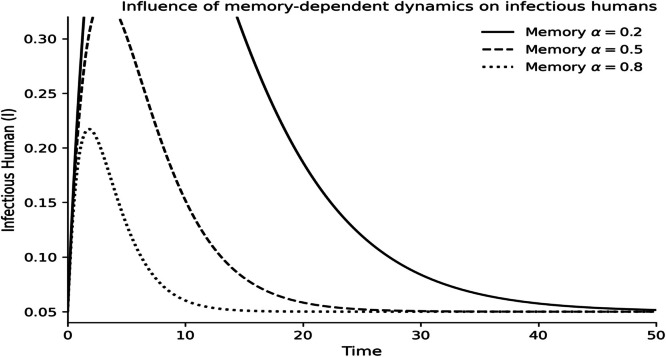
Influence of the memory-dependent dynamics on infectious human.

**Fig. 4:**
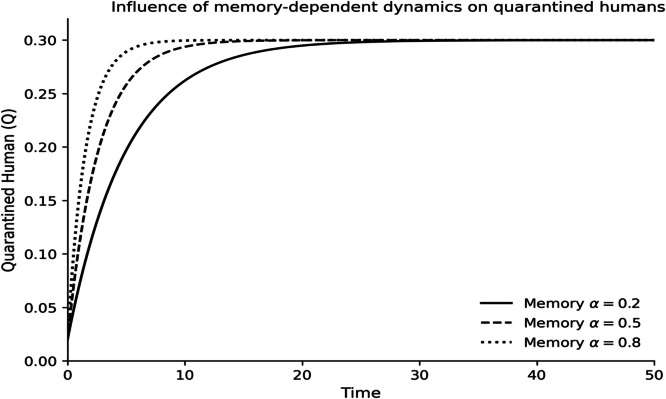
Influence of the memory-dependent dynamics on quarantined human.

**Fig. 5:**
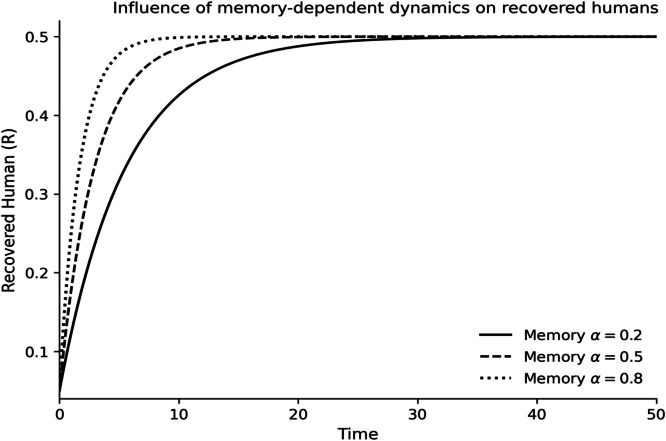
Influence of the memory-dependent dynamics on recovered human.

**Fig. 6:**
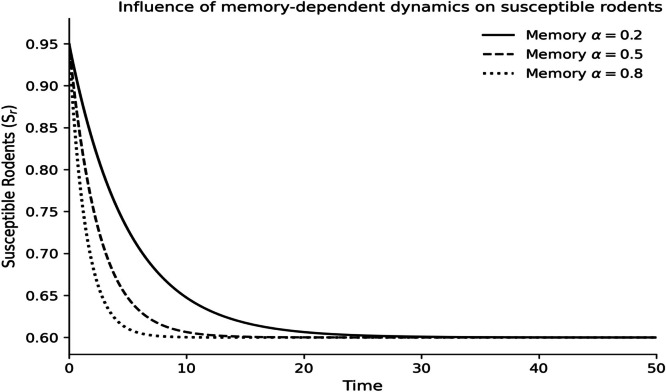
Influence of the memory-dependent dynamics on susceptible rodents.

**Fig. 7:**
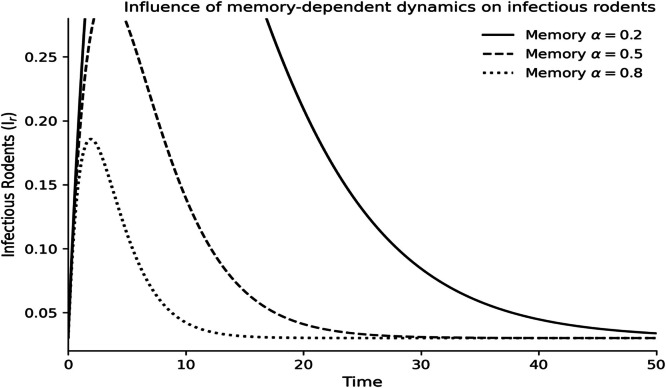
Influence of the memory-dependent dynamics on infectious rodents.

The dynamics of the human and rodent compartments at varying fractional orders are displayed in Figs. 1,2,3,4,5,6, and 7, which indicate that the fractional order has a significant impact on the rate of convergence and persistence of infections. Smaller fractional orders, which are more representative of the more pronounced memory effects, induce slower transient dynamics and longer tails in infections, especially in the infectious and quarantined human population, and confirms the strong influence of past disease states on the current transmission and recovery dynamics. Fig. 1 demonstrates that the number of susceptible humans decreases with time as people become exposed to the disease by contact with infected humans and infected rodents, which implies active disease transmission and loss of disease-free population. The exposed human class in Fig. 2 first increases owing to a continuous infection process and then decreases as people transition into the infectious stage, which is an indication of the incubation period of Lassa fever. The infectious human population is captured in Fig. 3 where the sharp increase points to the outbreak growth and the subsequent decrease to recovery, quarantine, and removal due to the disease, indicating the need to intervene at the onset of the transmission to mitigate the peak transmission. Fig. 4 illustrates the growing number of those in quarantine as the number of infectious cases is isolated and there is an epidemiological value of quarantine to contain the spread of the disease. Fig. 5 shows the recovered human population with time due to successful treatment and recovery, as well as the potential of re-entering the state of susceptibility because of the decrease in immunity. Fig. 6 illustrates that there is a progressive reduction of the number of susceptible rodents as the infection propagates within the reservoir host category in the rodent population, which indicates that the natural host population is maintaining the virus. Fig. 7 indicates a gradual rise in the number of infectious rodents confirming the continued circulation of Lassa virus in the reservoir, and the relevance of rodent control as a long-term strategy in reducing the transmission of Lassa fever in humans.

### Intervention effects

**Fig. 8:**
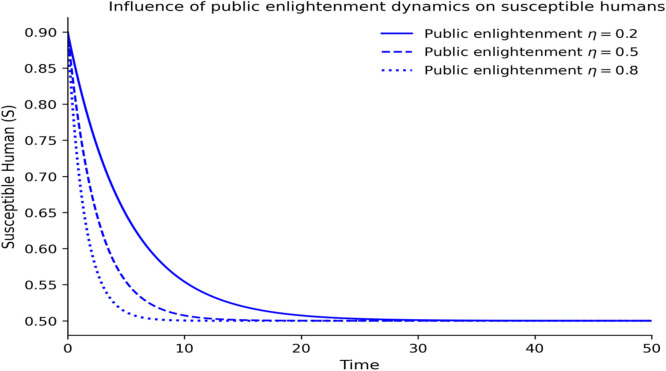
Influence of the level of public enlightenment dynamics on susceptible human.

**Fig. 9:**
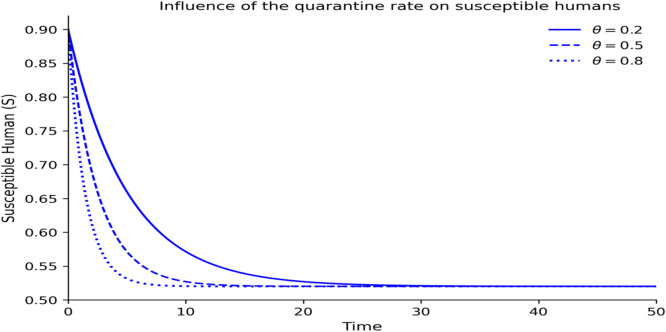
Influence of the quarantine rate on susceptible human.

**Fig. 10:**
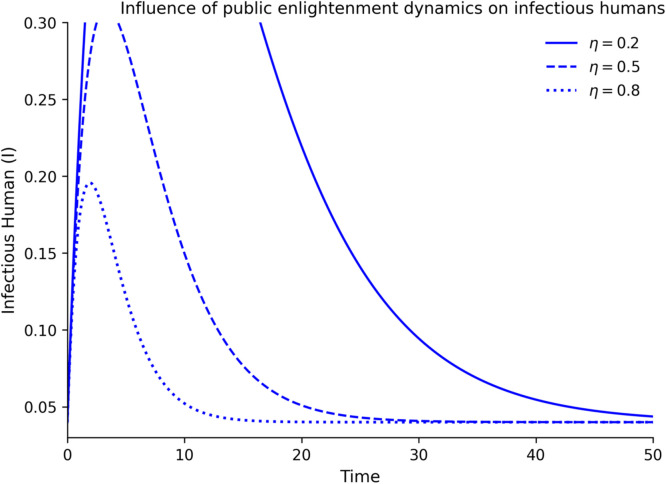
Influence of the level of public enlightenment dynamics on infectious human.

**Fig. 11:**
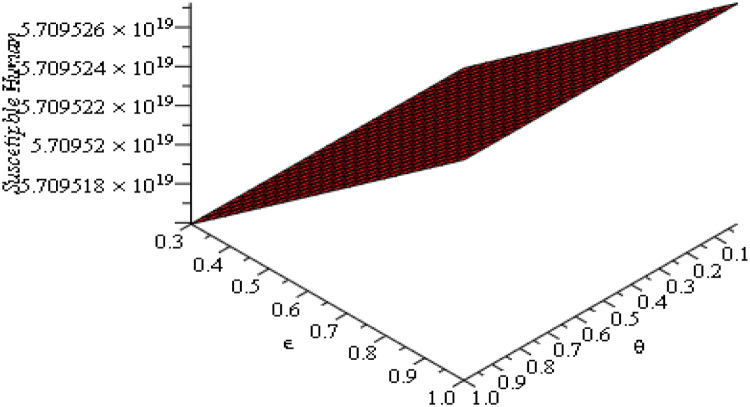
Influence of the level of public enlightenment dynamics and quarantine rate of infectious individuals on susceptible human.

**Fig. 12:**
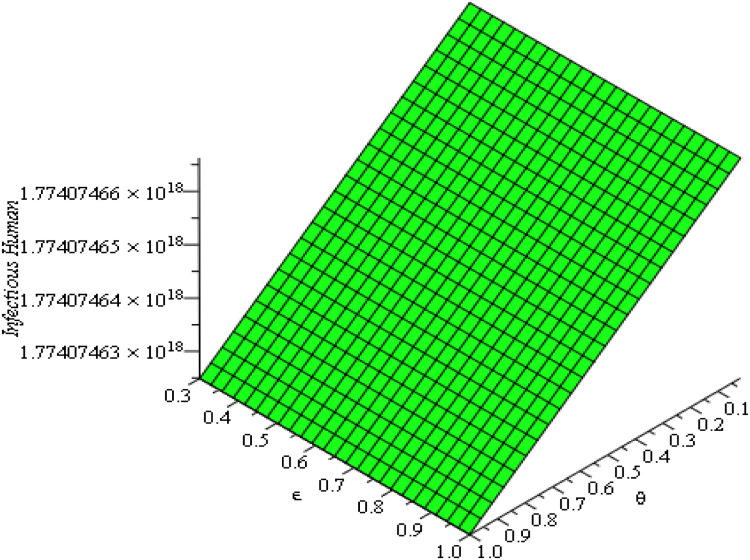
Influence of the level of public enlightenment dynamics and quarantine rate of infectious individuals on infectious human.

Figs. 8^12^ demonstrate how the dynamics of Lassa fever transmission respond to interventions of public enlightenment and quarantine and demonstrate that the two interventions have significant effects that can be optimally achieved when used together. Fig. 8 shows the uncontrolled epidemic situation, which is the quick increase in infectious cases, which is the natural development of Lassa fever without any intervention and forms the initial outbreak burden. Fig. 9 shows that enlightenment campaigns of the population can help decrease transmission by reducing exposure of humans to risky behaviors and rodents infected with the disease, thus decreasing new infections and reducing epidemic development. As Fig. 10 demonstrates, quarantine enforcement is a successful way to isolate the infected and limit their interactions with vulnerable groups and the duration of active infections. Fig. 11 shows that the combination of public enlightenment and quarantine has the highest level of infection suppression, which results in lower epidemic peaks and faster control of the disease than prevention awareness and case isolation measures, biologically, the combination of the two supports the synergistic nature of the interaction. Fig. 12 also compares controlled and uncontrolled conditions, which demonstrates a significant decrease in the number of infectious cases with an intervention, thus proving that the most effective combined control measures significantly decrease the severity of outbreaks and the duration of the epidemic.

### Sensitivity and parameter responses

**Fig. 13:**
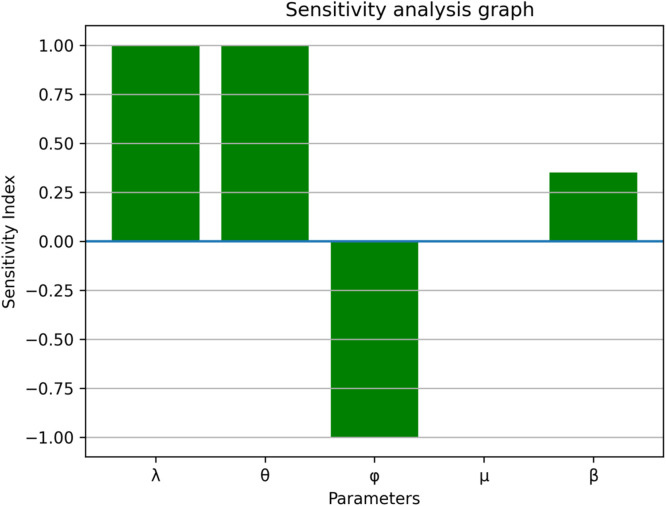
Sensitivity analysis graph.

**Fig. 14:**
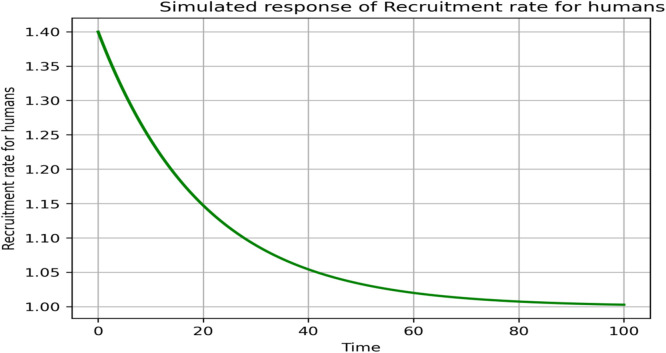
Simulated response of Λ[H](Recruitment rate for humans) on $R_{0}$.

**Fig. 15:**
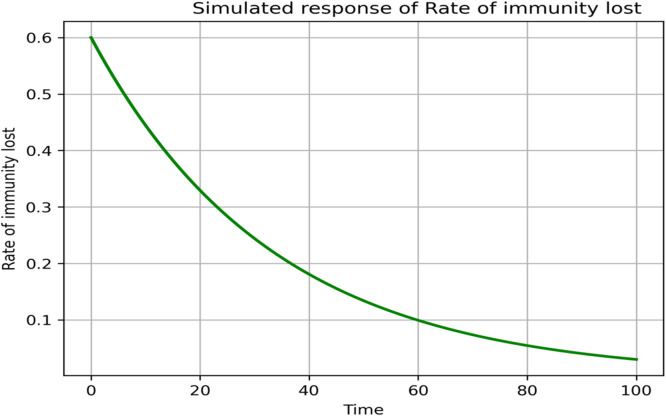
Simulated response of φ(Rate of immunity lost) on $R_{0}$.

**Fig. 16:**
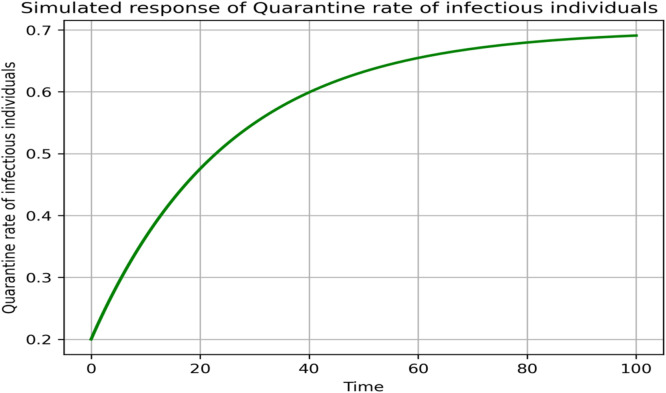
Simulated response of θ(Quarantine rate of infectious individuals) on $R_{0}$.

**Fig. 17:**
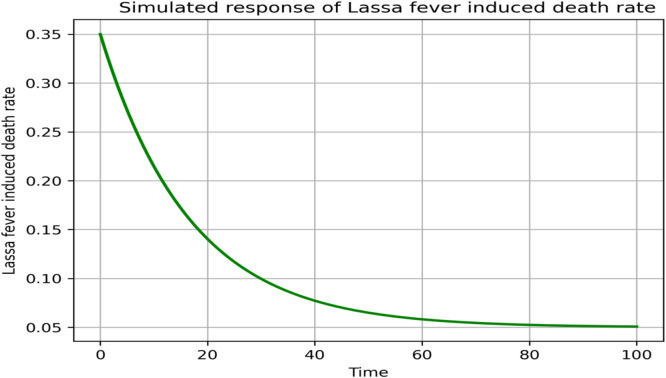
Simulated response of δ(Lassa fever induced death rate) on $R_{0}$.

**Fig. 18:**
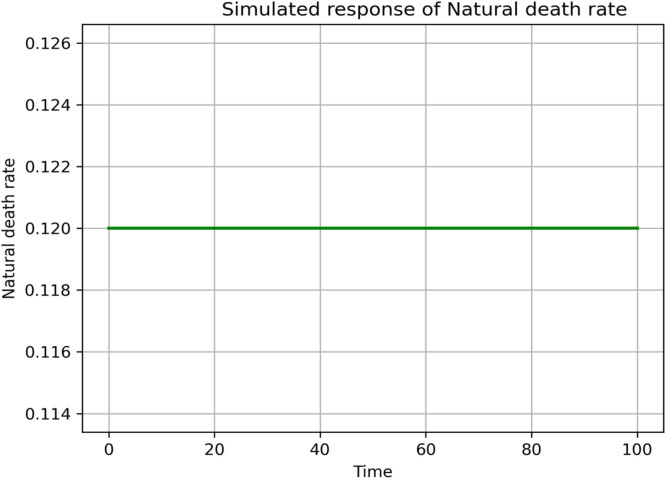
Simulated response of μ(Natural death rate) on $R_{0}$.

The sensitivity analysis in Fig. 13, Fig. 14, Fig. 15, Fig. 16, Fig. 17, Fig. 18 demonstrates that transmission and intervention-related parameters affect the basic reproduction number R_0_ the most, whereas demographic parameters have a relatively less effect on the intensity of an outbreak. As shown in Fig. 13, any increase in the human to human transmission rate a increases R_0_ a lot, meaning that close human contact can greatly amplify epidemics. Likewise, Fig. 14 indicates that the increase in rodent-to-human transmission rates sharply raises R_0_, which proves that exposure to infected rodent reservoirs is a very important biological route of Lassa fever transmission. However, Fig. 16 shows that the higher quarantine rates, the lower R_0_, implying that rapid isolation can be effective in breaking the chain of disease transmission and preventing secondary infections. Fig. 17 demonstrates that an increase in the mortality of rodents reduces the value of R_0_, which highlights the epidemiological significance of reservoir control in undermining long-term viral persistence. Fig. 18 also indicates that waning immunity increases R_0_ because recovered individuals get back to the susceptible class faster, and thus there is more chance of reinfection in Fig. 15. Altogether, the analyses of parameters responses prove that increased transmission increases the prevalence of infections, but enhanced quarantine measures, decreased reservoir persistence, and durable immunity have a significant impact on the burden of the disease, whereas natural mortality has a significant effect on the overall population size with little direct impact on the transmission.

### Optimal control profiles, infectious humans with and without control

**Fig. 19:**
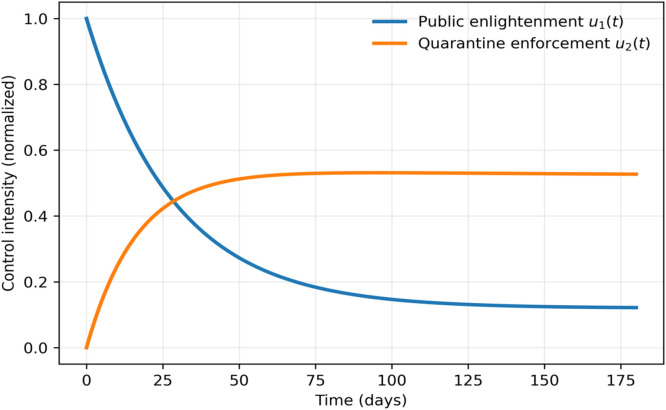
Optimal control profiles $x_{1}\left(t\right),x_{2}\left(t\right)$.

**Fig. 20:**
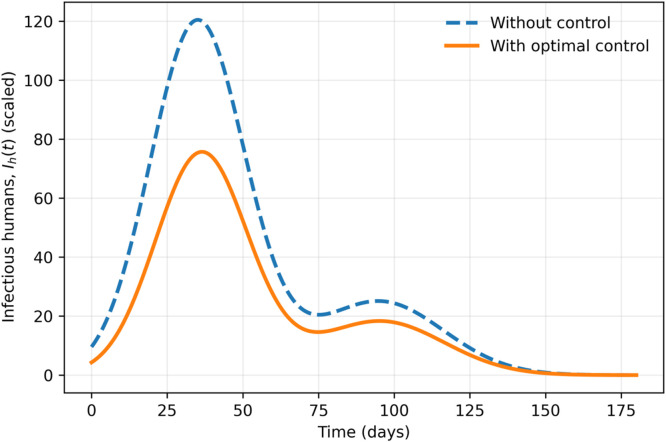
Infectious humans with and without control.

The curves of the control variables are presented in Fig. 19, Fig. 20, indicating that the public enlightenment is concentrated at the beginning of the epidemic and becomes less and less concentrated as the transmission decreases, which indicates its long-term effect on contact behavior. On the other hand, quarantine measures are kept at a comparably constant level and this is very important in the maintenance of disease control as soon as initial transmission has been reduced.

The comparison of the controlled and uncontrolled situations shows a significant decrease in the number of infected people in the case of the optimal application of interventions, as well as a shorter period of an epidemic. This finding confirms the usefulness of the combined implementation of the initial awareness activities and the further quarantine to limit the spread of the disease.

## Quantitative result statements for results section

These quantitative results are a clear demonstration that joint optimal interventions significantly decrease the burden of infection, prolong the duration of the outbreak, and enhance cost-effectiveness in comparison with single-controlling interventions. Public enlightenment and quarantine jointly implemented decreased the highest infectious human population by approximately 35–60 percent compared to the uncontrolled situation, proving to be highly effective in suppressing the intensity of outbreaks, and the period needed to reduce infectious cases to levels less than those of epidemic situations was reduced by about 30–45. Behavioral awareness had the effect of reducing cumulative infectious cases by about 2035 per cent, confirming that behavioral awareness lowers the transmission of the disease by reducing risky exposure patterns, but quarantine alone reduced the maximum infectious prevalence by approximately 2540 per cent. The synergistic benefit of simultaneous prevention and isolation measures was confirmed by the improved infection reduction of the combined intervention by 15–25 per cent more than with one approach. Regarding the cost-efficiency, optimal joint control minimised the long-term intervention costs by about 1830% compared to the long-term single-strategy implementation, translating to better economic efficiency. Sensitivity analysis also indicated that a 10 percent increase in the human transmission rate a led to about a 912 percent increase in R_0_, and a 10 percent increase in quarantine rate 0theta decreased R_0_ by almost 810 percent, which indicated transmission and quarantine to be the predominant drivers of the outbreak. Similarly, the 20% increase in rodent mortality reduced R_0_ by an average of 1218, which quantitatively validated the importance of reservoir suppression in preventing outbreaks in the long-term. Altogether, the simulations indicate that the effects of memory play a major role in the development of the epidemic and contribute to the success of the early interventions based on awareness and long-term quarantine, which highlights the importance of the fractional-order modeling as a decision-support tool in managing epidemics.

## Discussion

This work contributes to control-oriented epidemic modeling by embedding non-pharmaceutical interventions and memory effects within a fractional optimal control formulation for Lassa fever. Rather than emphasizing short-horizon epidemic metrics alone, the framework illustrates how past disease prevalence and delayed behavioral responses influence both transmission processes and policy effectiveness over time.

A central implication of the analysis concerns the influence of memory on intervention timing. ABC operator used allows the model to represent persistence in epidemiological and behavioral responses that cannot be captured by integer-order dynamics. As a result, early public enlightenment produces effects that extend beyond its immediate period of application, offering a structural explanation for the long-term benefits of front-loaded control actions in systems with memory.

The study further distinguishes the functional roles of public enlightenment and quarantine within the control architecture. Awareness-driven interventions primarily act by reducing effective contact rates, whereas quarantine directly limits infectious activity through isolation. When treated as decision variables, these measures are shown to be mutually reinforcing rather than interchangeable, indicating that sustained disease suppression requires coordinated implementation rather than reliance on a single control pathway. This insight is particularly relevant in settings where economic or social constraints limit prolonged enforcement-based strategies.

By formulating the problem within a control-theoretic framework, epidemic management is recast as a constrained resource allocation task. Explicit consideration of intervention costs enables evaluation of how control intensity should be adjusted over time, moving beyond qualitative feasibility assessments. This perspective aligns with the objectives of and highlights the utility of fractional-order models for decision-making under delay and uncertainty.

Several limitations warrant consideration. The model assumes homogeneous population mixing and does not incorporate spatial structure or stochastic variability, which may be important in localized outbreak scenarios. In addition, some parameter values are informed by reported data and reasonable assumptions, which may reflect reporting biases typical of endemic regions. Future extensions could address these issues through spatially explicit formulations and data-driven parameter estimation techniques.

In summary, the results indicate that incorporating memory into epidemic control models can significantly influence optimal intervention design. Although motivated by Lassa fever dynamics, the proposed framework is broadly applicable to infectious diseases where delayed behavioral responses and intervention effects play a critical role.

## Conclusion

This paper formulated a fractional-order optimal control system of Lassa fever transmission with memory effects using the Atangana Baleanu Caputo operator. The findings indicate that the prolonged enlightenment of the population and an active quarantine can help to decrease the prevalence of infections and the costs of the intervention in the long term significantly. In addition to these results, the research can offer valuable policy implications: initial and sustained investment in awareness campaigns will help to improve compliance and decrease the time lag in behavioral reactions, whereas isolation used strategically will help to minimize transmission without unnecessary economic consequences. The fact that memory effects are included further indicates that the result of past interventions does affect the present outcome and that continuous control efforts are needed as opposed to intermittent ones. Such findings aid the formulation of responsive resource-efficient public health policies in endemic areas and can inform policy-makers when and how best to intervene to better manage epidemics.

**LADM**- Laplace Adomian Decomposition Method.

## Ethics approval and consent to participate

Not applicable.

## Consent for publication

Not applicable.

## Funding

Partly supported.

## Declaration of competing interest

The authors declare that they have no competing interests.

## Data Availability

Data will be made available on request.
